# Understanding and experience of adverse event following immunization (AEFI) and its consequences among healthcare providers in Kebbi State, Nigeria: a qualitative study

**DOI:** 10.1186/s12913-022-08133-9

**Published:** 2022-06-03

**Authors:** Semeeh Akinwale Omoleke, Biniam Getachew, Abubakar Isyaku, Abdulrasheed Bello Aliyu, Ashiru Mohammed Mustapha, Shafiu Muhammad Dansanda, Kehinde Kazeem Kanmodi, Hafsat Abubakar, Zahraddeen Ibrahim Lawal, Haruna Abdullahi Kangiwa

**Affiliations:** 1 Doctoral Programme in International Public Health, Euclid University, Bangui, Central African Republic; 2 STOP Consultant, Stop Transmission of Polio (STOP) Program, Birnin Kebbi, Nigeria; 3 Veterinary Public Health, Ministry of Animal Health, Husbandry and Fisheries, Birnin Kebbi, Nigeria; 4 USAID Integrated Health Program, Kebbi State, Birnin Kebbi, Nigeria; 5 The Children International, Breakthrough Action Nigeria Project, Birnin Kebbi, Nigeria; 6 Child Health and Wellbeing (CHAW) Program, Cephas Health Research Initiative Inc, Ibadan, Nigeria; 7 Health Safety and Environment Department, Nigerian Pipelines and Storage Company, Nigerian National Petroleum Corporation, Abuja, Nigeria; 8 Programme Section, United Nations Children’s Fund (UNICEF), Kano, Nigeria; 9 Immunization and Disease Control Department, Kebbi State Primary Health Care Development Agency, Birnin Kebbi, Nigeria

**Keywords:** Adverse event following immunization (AEFI), Healthcare provider, Understanding and experience, Nigeria

## Abstract

**Background:**

Vaccines used in the national immunization program are relatively safe and effective. However, no vaccine is perfectly safe. Therefore, adverse reactions may occur. This study aimed to investigate the understanding and experience of Adverse Event Following Immunization (AEFI) among healthcare workers and Routine Immunization (RI) officers.

**Methods:**

Phenomenological qualitative study was conducted between June and September 2019, using a semi-structured question guide in Kebbi State, Northwest Nigeria. Face-to-face interviews were conducted with 12 RI providers, eight Expanded Program on Immunization (EPI) officers, and eight Disease Surveillance and Notification Officers. Thematic analysis was used to analyze the data. The interviews were transcribed and translated, then manually analyzed thematically.

**Results:**

The knowledge level of healthcare providers on AEFI definition and classification varied and was suboptimal. Error during vaccination was the study participants' most frequently mentioned possible cause of AEFI. Persistent crying, fever, fainting, and swelling and tenderness at injection sites were the AEFI experienced by the healthcare providers in their careers. Block rejection, lower immunization uptake, loss of confidence in RI, attack on RI providers, discrimination of RI providers and divorce threats among spouses were the consequences of AEFI. Supportive supervision of the RI sessions, refresher training on safe injection for RI providers, and symptomatic treatment of clients with AEFI would prevent AEFI consequences. Also, educating caregivers, community sensitization, and dialogue would minimize the consequences of AEFI.

**Conclusions:**

Evidence of a sub-optimal understanding of AEFI was established in this study. Hence, policymakers should consider regular refresher training on AEFI to ensure all RI providers have an optimal understanding of AEFI. Health education of caregivers and parents during RI sessions and community engagement should be considered to minimise AEFI consequences on the immunization program and the society.

**Supplementary Information:**

The online version contains supplementary material available at 10.1186/s12913-022-08133-9.

## Background

A vaccine is a vital public health tool to avert infectious diseases. Vaccines used in the national immunization program are safe and effective. However, no vaccine is perfectly safe, therefore, adverse reactions may occur [1]. According to the definition provided by World Health Organization (WHO), “Adverse Event Following Immunization (AEFI) is any untoward medical occurrence which follows immunization and which does not necessarily have a causal relationship with the usage of the vaccine.” The adverse event may be any unfavorable or unintended sign, abnormal laboratory finding, symptom, or disease. Reported adverse events can either be true adverse events, i.e., resulting from the vaccine or immunization process – or coincidental events that are not due to the vaccine or immunization process but are temporally associated with immunization [1]. Countries could adapt their own contextualized AEFI definition [2]. However, Nigeria follows what WHO recommended [3]. Regarding the causes of AEFI, it could stem from the five broad categories of AEFI as per WHO classification. These are vaccine-product related reaction; vaccine quality defect-reaction; immunization-related reaction; immunization-anxiety related reaction and coincidental reaction/event [1].

The Nigerian Demographic and Health Survey conducted in 2018 revealed that 31% of children aged 12–23 months had received all essential vaccinations, and 21% had received age-appropriate vaccination [4]. Different factors might have contributed to this low vaccination coverage, and AEFI could be one of them [5]. Improving the knowledge and practical skills of health care providers could minimize AEFI and increase client satisfaction, especially among families with vaccine hesitancy and low vaccination coverage areas [6]. Globally, in 2015, 60% of countries in WHO Region of Americas reported at least 10 AEFI per 100,000 surviving infants, 55% in European Region, 43% in Eastern Mediterranean Region, 33% in Western Pacific Region, 27% in South-East Asian Region and 21% in African Region [7]. In Nigeria, the prevalence of AEFI varies from 35% in Kano, Northwestern Nigeria, to 42% in Benin City of Southern Nigeria [8, 9].

Exploring the knowledge of AEFI among health care providers would provide evidence on actual practice and help strengthen the AEFI surveillance program. A study done in Zimbabwe revealed that none of the 61 interviewed nurses defined AEFI correctly. Besides, AEFI notification and investigation forms were available at six out of 18 health facilities [10]. There is far too little research conducted in Nigeria related to AEFI, yet very few assessed the knowledge, practice, and management of AEFI among health care providers. A study in Lagos reported that healthcare providers were fairly knowledgeable on the clinical spectrum and several aspects of AEFI [11]. Another interventional study done in Ilorin, Northcentral part of Nigeria, showed that most of the study participants had poor knowledge of safe immunization injection techniques and were unaware of any policy on injection safety before the health education intervention [12]. On the contrary, a study done in the Northern part of Nigeria portrayed that health care providers had good knowledge and perception of AEFI surveillance [13]. Therefore to improve and standardize the understanding, knowledge, and practice of AEFI among health care providers, the Federal Ministry of Health Nigeria developed an AEFI guideline introduced into Expanded Program on Immunization (EPI) a few years back [3].

In the 2017/18 measles vaccination campaign, Kebbi State reported the highest incidence of suspected AEFI, 101.3/100,000 population, compared with other States [14]. However, the AEFI report from RI was suboptimal. In 2018, 2019, and 2020 respectively, 211, 2288, and 4980 cases of AEFI were reported across the 21 Local Government Areas (LGAs) in Kebbi State.

This study is germane given the paucity of research on AEFI in Nigeria. Further, no previous work has been done in Kebbi State to assess the understanding and the experience of health workers despite the high incidence of AEFI reported from the measles mass vaccination campaign in 2017/18. Hence, this study aimed to investigate the understanding and experience of AEFI among healthcare workers and routine immunization (RI) officers in Kebbi State, Nigeria. Further, the study intends to share the experiences of health workers and RI providers on various types of AEFI, managing AEFI crises, and the consequences on RI uptake. Consequently, the findings from this study would provide empirical evidence to inform policy and practice to improve vaccine safety and, ultimately immunization uptake.

## Methods

### Study period and design

The study was conducted from June to September 2019. Purposive sampling technique was employed since the targeted populations of interest were clearly identified within the immunisation and surveillance space. Grounded theory, an inductive reasoning approach, was used to eliminate bias and allow an in-depth understanding of AEFI general knowledge and experience among the EPI managers and RI providers.

### Study setting

The study setting is Kebbi State, which is in the Northwest part of Nigeria. The study area has an estimated landmass of 36,800 km^2^. Kebbi State has four traditional emirates, namely Gwandu, Argungu, Yauri and Zuru. The State has 21 LGAs, which comprise one tertiary hospital, 35 secondary hospitals, and 855 primary health care centers (PHC), out of which 660 are providing RI services. The State has an estimated total population of 4,965,722, with 198,629 under-one year and 1,092,459 women of child-bearing age. The administrative coverage for Penta 3 for 2018 was 97% and a high drop-out rate (DOR) of 14%, while Penta 3 coverage improved to 116% and DOR fell to 5.7% in 2019. There are concerns about the quality of administrative data for routine immunization in Nigeria, specifically in Kebbi State [15]. A Multiple Indicator Cluster Survey/National Immunization Coverage Survey 2016/17 was done in Nigeria, indicating a poor performance that led to the declaration of an emergency in the selected States, mostly in Northern Nigeria. Kebbi State is one of the States declared under the State of Public Health Concern on Routine Immunization Programs by the National Primary Health Care Development Agency (NPHCDA), in collaboration with partners, due to low routine immunization (RI) performance [16].

### Study population

The study participants were health workers working at the LGA and health facility level, comprising Routine Immunization officers (otherwise called EPI managers), RI providers and disease surveillance and notification officers (DSNOs) from the Primary Health Care Department. In each emirate, at least seven study participants were recruited from each emirate for the interview. A total of 28 study participants were interviewed in this study. The inclusion criteria for the participation in this study were: being government personnel, at least three years of working experience in EPI or AEFI surveillance, and verbal consent to participate in the current study. Those who were sick at the time of the interview were excluded from the study.

### Data collection tools and methods

Before the outset of the data collection, at least three preparatory meetings were carried out among the researchers to develop the methodology and data collection tools. We used the purposive sampling method as earlier mentioned. There are four traditional emirates in Kebbi State, an average of 7 study participants were included from each emirate. There was an established prolonged engagement between the researchers and the study participants even before this research idea came out. The researchers were providing technical support in routine immunization space RI, which built the trust between the two parties. Data were gathered through in-depth interviews, observations in the field, and field notes using English- the official working language in Nigeria. A semi-structured open-ended question guide was used for all study participants. The average duration of the in-depth interviews generally was an hour, though some interviews lasted longer to allow the study participants fully express what they had in mind. Each respondent was provided with his/her interview transcription for validation before data analysis.

The interview guide contains questions arranged into the following themes: knowledge of AEFI and its classification, knowledge of possible causes of AEFI, experience on AEFI, consequences of AEFI, strategies adopted in educating caregivers on AEFI, AEFI related crisis ever witnessed, how the AEFI was managed and how to prevent AEFI crisis.

The semi-structured interview guide was generated from the literature review, theories and expert opinion of the research team. Data collectors were part of the research team and supported the study to the point of field data collection (interviews). Their level of involvement permitted rigor and a strong understanding of the essence of the study. The places of interviews were at their various places of work of the health workers and at their most conducive times. Each respondent was provided with his/her interview transcription for validation before data analysis. In addition to tape-recording, field notes were also taken to supplement the capturing of other expressions that could not be captured by the recordings. Finally, recruitment into the study was guided by the saturation point when no new information was generated from the interviews. This signaled the cessation of further recruitment into the study.

### Data analysis

Interviews were transcribed manually, and themes were generated from the manual transcription, which started with open coding across various research questions to generate answers based on their operational knowledge and understanding. The chunk of information generated was sorted and merged to create axial coding, which was grouped to create final themes across all the research questions.

## Results

As shown in Table 1, a total of 28 participants were recruited for the interview, which included Routine Immunization Officer (RIO) and DSNO from the LGA level and Routine Immunization providers at the primary healthcare level. Regarding the sex composition of the participants, seventy-five percent (75%) were males while 25% were female. All study participants were selected from the four traditional Emirates in Kebbi State.

**Table 1:** Demographic characteristics of the study participants

Variables	Categories	Number
Sex	Male	21
	Female	7
Emirates	Gwandu	5
	Argungu	3
	Yauri	10
	Zuru	10
Profession	Community Health Extension Worker	13
	Community Health Officer	7
	Environmental Health Officer	8
Role	RI provider	12
	RIO	8
	DSNO	8

The in-depth individual interview yielded ten (10) themes that were shared by healthcare workers, namely, knowledge of AEFI meaning/definition, its classification, knowledge of possible causes of AEFI, experience on AEFI, consequences of AEFI, strategies adopted in educating caregivers on AEFI, AEFI related crisis ever witnessed, consequences on immunization, how the AEFI was managed, and how to prevent AEFI crisis.

### Knowledge of AEFI and its classification

The knowledge of AEFI definition and classification was suboptimal among the study participants. Majority of the of the respondents simply defined AEFI as unfavorable reaction following immunization, a few defined it as immunization reaction, only one respondent defined it as adverse event following immunization that could be coincidental while two respondents could not explain or define AEFI. Some of the respondents were able to classify AEFI into two- mild and serious, while a few of them classified it as severe, moderate and mild. However, a minority of the respondents have no clue about AEFI classification.

*“AEFI is the reaction that follows immediately after immunization, there are two classifications, mild and serious AEFI. Mild AEFI most likely are fever, tenderness or redness at the site of injection. In terms of serious AEFI, there will be swelling at the leg, where you have to refer to the general hospital….”-*RT10

However, one of the respondents bluntly responded:

*“……AEFI is an adverse event that follows immediately after immunization, I don’t know the classification….”-*RT11

### Knowledge of possible causes of AEFI

Different possible causes or factors of AEFI were entertained by the respondents. The most frequently mentioned cause for AEFI was an error in vaccine administration. A respondent from a health facility said:

*“Due to the way the RI provider is administering the vaccine; lack of proper administration of the vaccine to the child may cause AEFI e.g., Penta vaccine given at the wrong site….”-*RT4

Vaccine defects, vaccine composition and vaccine overdoses were also mentioned as the possible cause of AEFI. Besides, anxiety and coincidental events were mentioned for the AEFI claim. A respondent shared his experience on a sick child who lost consciousness immediately after receiving a vaccine as follow:

*“It can be coincidental. I remembered during the yellow fever vaccination campaign, there was a girl down with fever before she fully recovered, the mother insisted that we have to administer a vaccine to the child, after receiving the vaccine, she immediately became unconscious, that was what led to AEFI, she was rushed to the hospital where after series of test and examination, she was said to have malaria”*-RT6

Another respondent attributed individual genetic make-up and vaccine recipient condition as a possible cause of AEFI.

*“Individual differences, the make-up of a person is one of the causes of AEFI, some people can eat “Tuwon Dawa” (cooked Guinea corn flour meal), and it becomes a problem while for others it’s normal, so as a result of the genetic make-up of an individual, they react to certain drugs”*-RT12

### Experience on AEFI

The study participants shared non-serious and serious AEFI encountered experiences they ever faced. Persistent crying, fever, faintness and swelling and tenderness at injection sites were among the frequently mentioned ones. A veteran respondent shared his experience on AEFI he has ever witnessed as follows.

*“I have experienced swelling, fever and redness, ....I have experienced coma, convulsion and shock….”* -RT20

Another respondent also explained that he has seen AEFI due to anxiety.

*“…. I have experienced a case of AEFI which was caused due to anxiety and because the child became afraid by seeing the syringe”-* RT24.

### Consequences and crisis of AEFI

The main consequences of AEFI stated by the respondents were lower immunization uptake and block rejection of vaccination. Due to the adverse events, families were rejecting the subsequent immunization doses and spreading misinformation that had happened to their child to the community members affecting the overall immunization uptake. Following AEFI, families would develop a negative perception of the immunization services, there could also be crises between spouses and boycotting other primary health care services. These were mentioned by some of the respondents.

*“I witnessed a caregiver refusing to allow her child to take the vaccine. When I went to investigate, the woman told me that her husband asked her not to allow them and if she refused, she would be divorced because the last time she took the child to receive vaccination, the child cried throughout the night”*-RT4

*“The consequences of AEFI on immunization were increase in drop-out rates and spread of rumors on Immunization Programs, …...if a serious AEFI occurs, especially in the villages, the story will spread easily, ….in that process, people will change the story and give it a different meaning and thereby causing rejection of immunization services”*-RT28

Out of the 28 respondents interviewed, only 7 reported an AEFI crisis. Four (4/7) respondents mentioned caregivers losing confidence in RI as the AEFI-related crisis witnessed. Further, attacks on RI service providers, discrimination of RI providers from the community, and divorce threats among spouses were mentioned as a crisis after AEFI happened.

One of the study participants who experienced an attack by an aggrieved community as a result of the AEFI crisis shared his experience below;

*“I had an experience when we went to a community, the people in that community chased us away with garden-hoes telling us not to touch any child because the last injection, …. the legs of their children were weak”*-RT11

### Strategies adopted in educating caregivers on AEFI

Three broad sub-themes emerged from the analysis of the strategies adopted in educating caregivers on AEFI, which include educating caregivers, community sensitization and community dialogue. The respondents stated that educating caregivers one-to-one during and after vaccination about the possible clinical event that could follow the use of specific vaccines was expressed by the respondents to prevent misinformation about the vaccines. Beyond that, community sensitization and dialogue and reassurance of caregivers were strategies mentioned to overcome community vaccine hesitancy following AEFI.

*“One of the strategies we adopt is sensitization of the community on AEFI”*-RT8

*“ …. with the help of the community leader, we resolved the AEFI problem”*-RT11

*“We need to tell them the possibilities of AEFI happening and how they can manage it or report back to the health facility. Health educating the caregivers will make them not to panic when AEFI occur to their children because they were already informed”*-RT24

### How can we prevent AEFI?

The respondents mentioned how to prevent AEFI crisis, such as caregivers’ education, refresher training for RI service providers, supportive supervision, maintaining safe injection procedure, and symptomatic treatment for children.

“*…. I told the RI provider to health educate the caregivers before conducting any session on the issue of AEFI and I educated the RI provider on how to educate caregivers during RI”-*RT5

*“Before we can prevent something, we must know the cause . . . in terms of overdosing, the health worker had to be trained and at the same time, the site of injection, the wrong site, still the health workers must be trained very well to be able to tackle all these problems”-*RT18

## Discussion

The most obvious findings that emerged from this study are the varying understanding of AEFI definition and classification among RI providers. Among different possible causes of AEFI, error during vaccination was frequently mentioned by the study participants. Low uptake of immunization due to AEFI was one of the consequences of AEFI at the community level. Educating caregivers, community sensitization and community dialogue were actions taken by the respondents to overcome the consequence of AEFI. Besides, refresher training on safe injection procedures and symptomatic treatment of AEFI were ways to prevent AEFI and its negative consequences. In this study, the understanding and knowledge of the AEFI definition by healthcare providers were suboptimal. A study in Zimbabwe found a gap in knowledge where none of the health workers could precisely define an AEFI[10].

Another study conducted in Kaduna State, Nigeria, revealed that close to 60% of the healthcare providers had good knowledge of AEFI [13]. The finding of the study is similar to our study. However, our study varied from the Kaduna study due to a methodological difference in measuring the AEFI knowledge. The Kaduna study used scores to assess AEFI knowledge, then categorized it as good and poor knowledge [13]. In our study, we used the qualitative method and the study participants were expected to define according to the AEFI guideline. A study in Ghana also reported that 83.4% of healthcare professionals correctly defined AEFI [18]. However, this study from Ghana assessed the general knowledge of AEFI by asking the meaning of the acronym ‘AEFI’, which differs in the measurement of AEFI definition in our own study.

The Nigerian AEFI surveillance guideline broadly categorized AEFI based on causes, seriousness and frequency. Cause-specific types of AEFI are classified as vaccine product-related reaction, vaccine quality defect-related reaction, immunization error-related reaction, immunization anxiety-related reaction and coincidental events. Vaccine reactions by seriousness and frequency are classified as non-serious reactions and serious reactions [17]. Most of the study participants in our study were not able to classify AEFI as per the guideline.

In our study, the most repeatedly encountered AEFI types were immunization error-related and coincidental causes of AEFIs. However, one respondent mentioned genetic make-up as a possible cause of AEFI, which was not mentioned in the guideline. This suggests a suboptimal understanding of possible causes of AEFI among RI providers.

All the study participants in our study had experienced either non-serious or serious or both types of AEFI. A study done in Lagos, Nigeria, stated that 33.5% of healthcare workers directly involved with vaccination and management of AEFI had encountered an AEFI [11]. The difference between our study and the Lagos study might be due to study participant selection. Our study included healthcare providers with at least three years of RI service provision. Our study is in tandem with the Australian study, where most of the study participants experienced suspected AEFI in their careers [19]. The AEFI cases experienced by the study participants in Australia were suspected hypotonic hypo-responsive events, anaphylaxis, febrile convulsion, non-febrile convulsions, extensive limb swelling, high fevers and skin rashes [19]. Whereas, most frequently mentioned clinical features by respondents in our study were persistent crying, fever, fainting attack and swelling and tenderness at injection sites. The possible difference between the suspected AEFIs could be due to the difference in medical technology used to classify the AEFIs between Australia and Nigeria, and the variation in clinical acumen of the health workers.

Block rejection, lower immunization uptake, loss of confidence in RI, attack on RI providers, discrimination of RI providers, and divorce threats among spouses were the consequences and crises the healthcare providers mentioned due to AEFI. Proactive and reactive communication with the caregivers and community members would minimize the consequences of AEFI [17]. In line with the guideline, the respondents in our study explained that health education of the caregivers, community sensitization, and community dialogues were strategies adopted to overcome AEFI consequences.

To prevent the consequence of AEFI due to error from the RI provider, the participants in our study mentioned close supportive supervision of the RI sessions, refresher training on safe injection for RI providers and symptomatic treatment for clients with AEFI would be helpful. A study done in Kaduna State, Nigeria, stated that most of the RI providers expressed the need for training and retraining on AEFI [13].

One of the strengths of this study was the qualitative approach which allowed the RI providers to share their AEFI experience in detail and depth. However, this study has a few limitations. First, we selected the study participants purposively at the PHC level, where most RI activities are conducted. This purposive selection excluded the healthcare providers at the secondary and tertiary levels. However, less than 5% of all RI activities occur at the secondary and tertiary health facilities altogether in Kebbi State. Therefore, this exclusion is unlikely to impact the study findings. Second, since we studied the lifetime experience of AEFI among healthcare providers, the study might be affected by recall bias. Finally, the experience of reporting and documenting AEFI cases was not included in this study. This could be a subject of future investigation.

## Conclusion

There was a general knowledge gap in AEFI definition and classification among healthcare providers. Insights were provided regarding the ramifications of the AEFI on RI uptake in the study setting. Therefore, we recommend that policymakers consider regular refresher training for healthcare providers on AEFI. This will minimize the occurrence and prevent the unintended consequences of AEFI. Besides, health education to caregivers during RI sessions, community engagement and sensitization should be strengthened to avoid low uptake of immunization due to AEFI.

## Supplementary Information

**Additional file 1.**

## Data Availability

The relevant data analyzed during the study are included in the published manuscript.
