# Correction: Understanding and experience of adverse event following immunization (AEFI) and its consequences among healthcare providers in Kebbi State, Nigeria: a qualitative study

**DOI:** 10.1186/s12913-022-08252-3

**Published:** 2022-07-07

**Authors:** Semeeh Akinwale Omoleke, Biniam Getachew, Abubakar Isyaku, Abdulrasheed Bello Aliyu, Ashiru Mohammed Mustapha, Shafiu Muhammad Dansanda, Kehinde Kazeem Kanmodi, Hafsat Abubakar, Zahraddeen Ibrahim Lawal, Haruna Abdullahi Kangiwa

**Affiliations:** 1 Doctoral Programme in International Public Health, Euclid University, Bangui, Central African Republic; 2 STOP Consultant, Stop Transmission of Polio (STOP) Program, Birnin Kebbi, Nigeria; 3 Veterinary Public Health, Ministry of Animal Health, Husbandry and Fisheries, Birnin Kebbi, Nigeria; 4 USAID Integrated Health Program, Birnin Kebbi, Kebbi State Nigeria; 5 The Children International, Breakthrough Action Nigeria Project, Birnin Kebbi, Nigeria; 6 Child Health and Wellbeing (CHAW) Program, Cephas Health Research Initiative Inc, Ibadan, Nigeria; 7 Health Safety and Environment Department, Nigerian Pipelines and Storage Company, Nigerian National Petroleum Corporation, Abuja, Nigeria; 8 Programme Section, United Nations Children’s Fund (UNICEF), Kano, Nigeria; 9 Immunization and Disease Control Department, Kebbi State Primary Health Care Development Agency, Birnin Kebbi, Nigeria

**Correction to: BMC Health Serv Res 22, 741 (2022)**

**https://doi.org/10.1186/s12913-022-08133-9**

Following publication of the original article [1], the authors identified an error in the affiliations assignment: affiliation 1 (Doctoral Programme in International Public Health, Euclid University, Bangui, Central African Republic) was incorrectly assigned to Abdulrasheed Bello Aliyu and Zahraddeen Ibrahim Lawal.

This error is corrected in the author list of this Correction article and the original article [1] has been updated.
