# Supplementary material for: Understanding and experience of adverse event following immunization (AEFI) and its consequences among healthcare providers in Kebbi State, Nigeria: a qualitative study
Source: BMC Health Serv Res. 2022 Jun 3;22:741. doi: 10.1186/s12913-022-08133-9 (PMC9166581; doi:10.1186/s12913-022-08133-9)
Supplement: Supplementary file 1 — Additional file 1. [file 12913_2022_8133_MOESM1_ESM.docx]

| **S/N** | **Issue** | **Open codes generated** | **Mention by** | **Frequency of codes generated** | **Axial codes generated** |
| --- | --- | --- | --- | --- | --- |
| 1 | AEFI Definition | Adverse event following immunization | LIO2, DSNO4, RI-P12 | 17 | Unfavorable reaction following immunization |
|  |  | Problem that arises after immunization | RI-P | 1 |  |
|  |  | Unfavorable condition following immunization | RI-P5 | 5 |  |
|  |  | Reaction after immunization | DSNO, RI-P | 2 | Reaction from Immunization |
|  |  | Adverse event following immunization that is a co-incidental happening or occurrence | LIO | 1 | Adverse event following immunization that could be co-incidental |
|  |  | Have no idea | DSNO | 2 | have no idea |
| 2 | AEFI Classification | Mild and serious  minor and serious or major | LIO2 | **2** | Mild and serious |
|  |  |  | LIO2, DSNO6, RI-P8 | **16** |  |
|  |  | Severe, moderate and the others | RI-P | **1** | Severe, moderate and mild |
|  |  | Severe, moderate, mild | RI-P | **1** |  |
|  |  | severe and mild or minor | LIO2, RI-P2, RI-P | **5** |  |
|  |  | no idea about the classification | RI-P2 | **2** | No idea about the classification |
|  |  | Don’t know | RI-p | **1** |  |
| 3 | Possible causes of AEFI | Improper administration of vaccine | LIO4, DSNO7, RI-P8 | 19 | Error in vaccine administration |
|  |  | child is not held properly during administration | RI-P, LIO | 2 |  |
|  |  | personnel not well trained | RI-P2 | 2 |  |
|  |  | Use of contaminated syringes | RI-P | 1 |  |
|  |  | vaccine quality effects | LIO2, DSNO2 | 4 | Vaccine defects |
|  |  | vaccine has lost its potency | RI-P2, LIO | 3 |  |
|  |  | Improper handling of the vaccine | RI-P2 | 2 |  |
|  |  | Improper storage or management of vaccine | RI-P2 | 2 |  |
|  |  | Vaccine has expired | RI-P3 | 3 |  |
|  |  | Vaccine VVM 3-4 | RI-P3, DSNO | 4 |  |
|  |  | Using frozen vaccine | RI-P | 1 |  |
|  |  | Using wrong diluent | RI-P | 1 |  |
|  |  | Vaccine related | LIO, DSNO3, RI-P2 | 6 | Vaccine compositions |
|  |  | Antigens combination | RI-P | 1 |  |
|  |  | Action of antigens | DSNO | 1 |  |
|  |  | Sometimes anxiety | LIO2 | 2 | Anxiety |
|  |  | naturally scared of injection | LIO, DSNO | 2 |  |
|  |  | overdose of vaccine | RI-P | 1 | Vaccine overdose |
|  |  | it is newly introduced to your body system | DSNO | 1 | Genetic makeup |
|  |  | Genetic makeup | RI-P, LIO2 | 3 |  |
|  |  | co-incidental | DSNO3, RI-P2, LIO | 6 | co-incidental |
|  |  | Vaccinating child with fever | LIO | 1 | Recipient condition |
|  |  | not eating before receiving vaccine | DSNO | 1 |  |
|  |  | background sickness of the client | LIO | 1 |  |
|  |  | vaccination in the hot weather | LIO | 1 |  |
| 4 | Experience on AEFI | spent the night crying | LIO, RI-P | 2 | Troubling parents |
|  |  | Crying throughout the day | LIO, DSNO2 | 3 |  |
|  |  | leg swells up which goes away within two days. | RI-P2, DSNO | 3 |  |
|  |  | pain at injection site | LIO, RI-P | 2 | Mild health effect |
|  |  | High fever | DSNO, RI-P3 | 4 |  |
|  |  | tenderness | RI-P | 1 |  |
|  |  | Headache | LIO | 1 |  |
|  |  | Rashes | LIO | 1 |  |
|  |  | Block rejection | RI-P | 1 |  |
|  |  | Vomiting | LIO | 1 |  |
|  |  | Blister | DSNO | 1 |  |
|  |  | Fell down | RI-P, LIO | 2 | Serious health effect |
|  |  | Child having heavy hand | LIO | 1 |  |
|  |  | Coma | RI-P | 1 |  |
|  |  | convulsion | RI-P | 1 |  |
|  |  | Child fainted after vaccination | RI-P | 1 |  |
| 5 | Consequences of AEFI | discourage the mother on subsequent visits | LIO, dsno | 2 | Block rejection |
|  |  | Client dropout | DSNO2, RI-P2, LIO | 4 |  |
|  |  | Many people to shy away from immunization because of the fear of pain | RI-P | 1 |  |
|  |  | rejection of immunization services | Dsno | 1 |  |
|  |  | none of my children will be immunize again | DSNO, RI-P2 | 3 |  |
|  |  | Not able to do house work because of child crying | LIO | 1 | Family disturbances |
|  |  | Fever and excessive crying normally happen in Penta-1 & hardly experience with subsequent dose | RI-P3 | 3 |  |
|  |  | Not able to sleep | DSNO, RI-P | 2 |  |
|  |  | child will spend a day or two not being able to stretch his leg | RI-P | 1 | Mild health defect |
|  |  | spread of rumours on Immunization programs | DSNO | 1 | Negative perception on immunization services |
|  |  | rumours on Immunization programs | RI-P | 1 |  |
| 6 | How you educate caregivers on AEFI | we use to educate mother that vaccine is foreign to the body and it will stimulate antibodies and child will notice changes | RI-P | 1 | Community sensitization |
|  |  | tell mothers injection makes the child body temperature hot | DSNO | 1 |  |
|  |  | tell him the importance of the vaccine and what the vaccine prevents | DSNO | 1 | Educating caregivers |
|  |  | compare the importance OF Vaccine and the pain child undergo | DSNO, RI-P | 2 |  |
|  |  | Caregivers sensitization and health talk prior to beginning of immunization session | LIO2, DSNO, RI-P2 | 5 | Educating caregivers |
|  |  | Caregiver sensitization on the process of administering vaccine to a child | LIO2, RI-P3 | 5 |  |
|  |  | organizing meeting with community leaders | DSNO | 1 | Com dialogue |
| 7 | AEFI related crisis you have ever witness | community members lose confidence in RI service provider | RI-P | 1 | Losing confidence on RI p |
|  |  | massive community RI-P rejection | RI-P2 | 2 |  |
|  |  | caregiver taking child to black smith to puncture injection blisters convinced other caregivers to reject immunization | LIO | 1 |  |
|  |  | Community member chase away RI service provider with hoes | RI-P | 1 | Attack on RI provider |
|  |  | Divorce threat by husband | LIO | 1 | Divorce threat |
|  |  | Community head threaten to eject a defaulter from a community | RI-P | 1 | Threat to eject defaulters |
| 8 | what were the consequences | increased dropout rate in the community | LIO, RI-P3 | 4 | Drop immunization coverage |
|  |  | low coverage rate | RI-P2 | 2 |  |
|  |  | an outreach for hours without turns out | RI-P | 1 |  |
|  |  | block rejection | RI-P | 1 |  |
|  |  | defaulters | LIO | 1 |  |
|  |  | fight between spouses | LIO2 | 2 | Crisis between spouse |
|  |  | boycotting treatment and other PHC services | RI-P | 1 | Boycotting PHC services |
| 9 | How was it managed | collaboration with community leaders to arrest situation, | LIO, RI-P2 | 3 | Community dialogue |
|  |  | sensitization of community with community leaders | RI-P3, DSNO | 4 |  |
|  |  | household health talk using ward development committees | RI-P | 1 |  |
|  |  | sensitization of community stakeholders | RI-P | 1 | Community sensitization |
|  |  | investigation and reassurance of caregivers by supervisors | LIO | 1 | Supervisor’s intervention |
| 10 | How can we prevent it from happening? | give paracetamol to every child that takes pentavalent vaccine, | RI-P2 | 2 | Symptomatic treatment |
|  |  | ensures the child take the medicine immediately | RI-P | 1 |  |
|  |  | refresher training for RI service providers on how to conduct health education | LIO | 1 | Refresher training |
|  |  | continuous training of RI service providers | DSNO, RI-P2, LIO | 4 |  |
|  |  | RI information, education and communication materials paste in the house of community leaders, | RI-P | 1 | Community sensitization using IEC Material |
|  |  | Community sensitization | DSNO | 1 |  |
|  |  | Conducting caregiver’s health education on AEFI before sessions | LIO, RI-P5, DSNO | 7 | Educating caregivers |
|  |  | maintain proper injection safety procedure | RI-P2, DSNO | 3 | Safe injection procedure |
|  |  | intensify supportive supervision | RI-P2, LIO | 3 | Supportive supervision |
